# The Histone Deacetylase Inhibitor LBH589 (Panobinostat) Modulates the Crosstalk of Lymphocytes with Hodgkin Lymphoma Cell Lines

**DOI:** 10.1371/journal.pone.0079502

**Published:** 2013-11-21

**Authors:** Jan M. Klein, Alexander Henke, Maike Sauer, Martina Bessler, Katrin S. Reiners, Andreas Engert, Hinrich P. Hansen, Elke Pogge von Strandmann

**Affiliations:** Department I of Internal Medicine, Innate Immunity Group, University Clinic Cologne, Cologne, Germany; European Institute of Oncology, Italy

## Abstract

Epigenetic changes have been implicated in the malignant phenotype of Hodgkin Reed Sternberg (HRS) cells in Hodgkin lymphoma (HL), where HRS survival and proliferation depends on the microenvironment. The histone-deacetylase (HDAC) inhibitor LBH589 (panobinostat) showed clinical efficacy but its impact on the HRS microenvironment is unclear. Hence, we analysed the effects of LBH589 on lymphocytes and also potential combination therapies. In lymphocyte-target cell killing assays, LBH589-treatment triggered an enhanced lymphocyte-dependent lysis of HL cells despite of mild lymphocytopenic effects. In co-culture experiments of lymphocytes with HL cells, LBH589 suppressed the IFNgamma-release but increased the TNFalpha secretion. Recombinant TNFalpha boosted the lymphocyte-dependent lysis of HL target cells. In HL cell lines, LBH589 induced cell death, autophagy, and an increase of MICA/B that are ligands to natural killer cell receptors. The combination of LBH589 with Brentuximab Vedotin was inefficient due to down-regulation of CD30 as a target. Combination with gemcitabine revealed highly significant effects, suggesting a potential combination for future therapy. Based on these data we suggest that LBH589 favourably modulates the cytokine network and lymphocyte activity in the HL microenvironment.

## Introduction

In Hodgkin lymphoma (HL), the affected lymph nodes contain the malignant Hodgkin-Reed-Sternberg (HRS) cells, which are surrounded by and critically dependent on supporting, non-transformed lymphocytes, which in turn constitute the vast majority of the tumour mass [1]. Therefore, combined targeting of the tumour cells and their surrounding microenvironment is a promising strategy for the treatment of HL. The histone deacetylase inhibitor (HDACi) LBH589 (Panobinostat) is a new drug and demonstrated recently anti-tumour activity in relapsed HL patients in a phase II clinical trial [2]. However, its mechanism of action is not fully understood yet, especially in the interplay with lymphocytes.

Thus, the aim of this study was to examine the effect of LBH589 on primary lymphocyte function, including cytokine release, cytotoxicity and the interaction between HL cell lines and lymphocytes.

## Results and Discussion

We chose the widely accepted L428 and L540 Hodgkin lymphoma cell line, as these represent the B and T cell phenotype of classical HL, respectively [3]. A previous report showed that LBH589 affects identical pathways in L428, KM-H2 and HDLM-2 cell lines such as activation of caspases and mTOR pathways but inhibition of STAT5/6 [4]. This suggests strongly that LBH589 acts upon general HL cell features.

**Figure 1:**
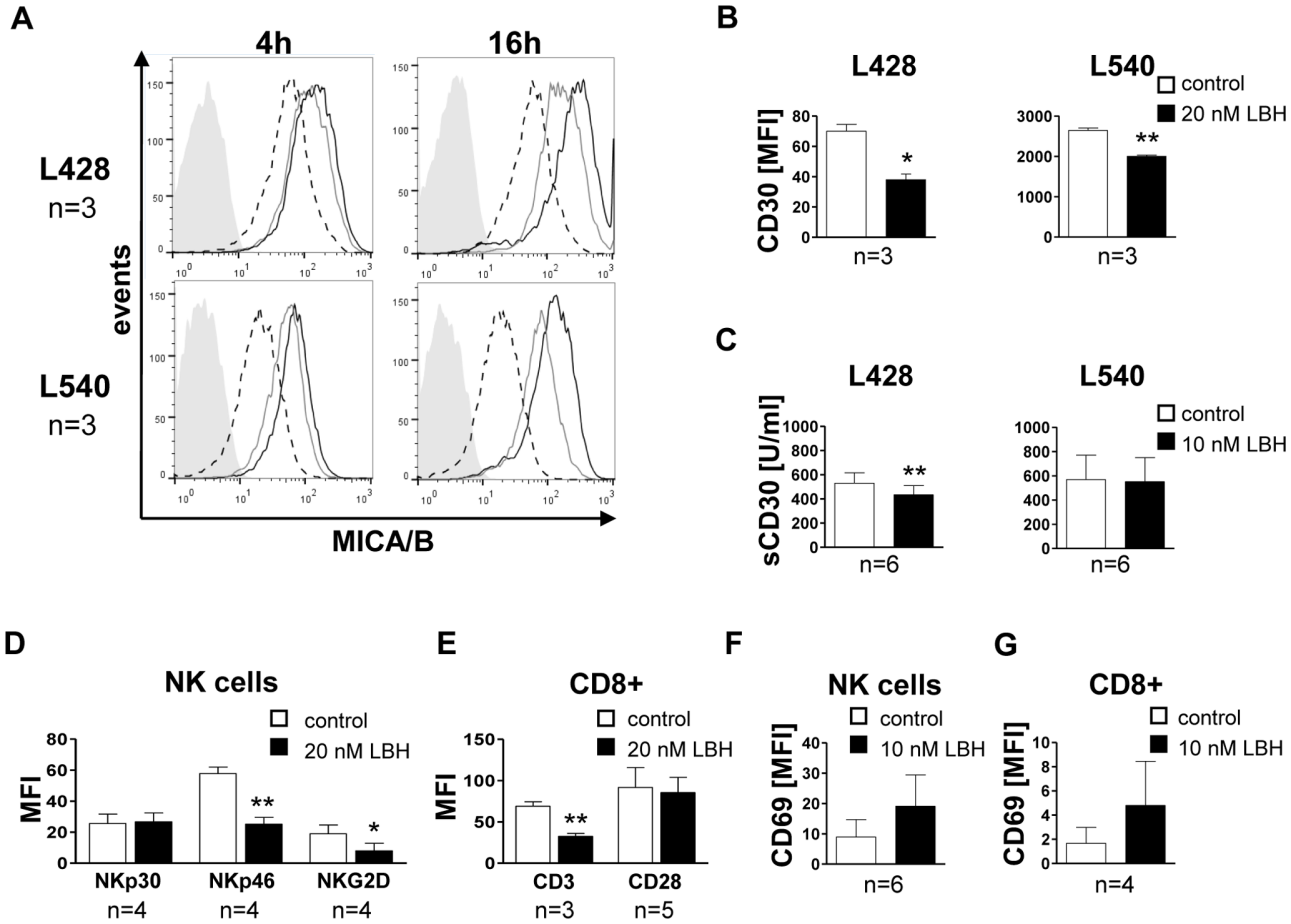
LBH589 modulated the expression of surface markers in cancer and immune effector cells. (**A**) Flow cytometry for the cell surface marker MICA/B on HL cells after 4 and 16 hours incubation time with 0, 10 or 20 nM LBH589. One representative experiment of three is shown. Shaded grey = IgG control; dashed line = 0 nM, solid grey line = 10 nM and black line = 20 nM. The isotype control experiment was performed in the presence of 20 nM LBH589. (**B**) The expression of CD30 on the surface of L428 and L540 cells was detected via flow cytometry. CD30 was significantly decreased after treatment with 20 nM LBH589 (48 h; n = 3; two-sided t-test). A representative histogram is shown in Fig. S1A in File S1. (**C**) Shed CD30 (sCD30) was measured via ELISA in cell culture supernatants. Upon treatment with 10 nM LBH589, sCD30 levels were significantly lower in L428 supernatants but not for L540 supernatants (t = 48 h; n = 6 per cell line; two-sided, paired t-test). (**D/E**) Flow cytometry to detect activating NK cell receptors (**D**) and T cell receptors (**E**). NK and T cells were treated for 24 hours with 20 nM LBH589. n = 4, two-sided, paired t-test. T cells: n = 3 for CD3 and n = 5 for CD28; two-sided, paired t-test. Representative histograms are shown in Fig. S1 D and E in File S1, respectively. MFI = mean fluorescent intensity. (**F/G**) The activation marker CD69 was upregulated on both, NK cells (**F**) and T cells (**G**) but not at statistically significant levels (p>0.05). Representative histograms are shown in Fig. S1 F in File S1.

First, we analysed the expression of ligands and receptors that play a role in the immune cell effector function on target and immune effector cells in response to LBH589. Treatment of HL cell lines L428 and L540 led to an upregulation of MICA/B on the cell surface (Fig. 1A). MICA/B are ligands for the activating receptor NKG2D, which is expressed on natural killer (NK) cells and T cell subsets. The surface expression levels of CD80, CD86, CD95 and CD262 did not change significantly (exemplary data in Figure S1C in File S1). This is in line with previous studies, in which treatment with different HDACi augmented the expression of NK cell activating ligands [5], [6]. CD30, a member of the TNF receptor superfamily, is overexpressed on HRS cells and known as a critical ligand-independent pro-survival factor [7]. The cell surface expression of CD30 was decreased after treatment with LBH589 (Fig. 1B). This was not caused by enhanced shedding, since CD30 in cell culture supernatants was also decreased (Fig. 1C). A downregulation of CD30 was also confirmed at the mRNA level (Fig. S1B in File S1) and is reported for the first time for LBH589 but was also shown for suberoylanilide hydroxamic acid (SAHA; vorinostat; a class I HDACi) [8].

Subsequently, we examined the effects of LBH589 on effector lymphocytes. NK cells showed a significant downregulation of the activating receptors NKp46 and NKG2D but not for NKp30 after 24 h of incubation (summary bar chart in Fig. 1D; a representative histogram of an experiment is shown in Supplementary Figure S1D). A previous study of the HDACi valproic acid (VPA) and SAHA indicated downregulation of NKp30, NKp46 and NKG2D but used higher doses and did not perform statistical analyses of results. Hence, a proper comparison to our results is difficult [9]. CD8+ T cells revealed a significant downregulation of the T cell receptor CD3, which is essential for T cell activation. The expression of the co-stimulatory receptor CD28 remained unchanged (summary bar chart in Fig. 1E; a representative histogram of an experiment is shown in Supplementary Figure S1E). Both, NK and CD8+ T cells, showed increased expression of the activation marker CD69 but this increase did not reach the level of statistical significance due to high intra-group variance (summary bar charts in Fig. 1F/G, respectively; representative histograms of experiments are in Supplementary Figure S1F/G).

We then examined immune cell effector functions. First we studied how LBH589 influenced the release of pro-inflammatory cytokines as essential component of the HL microenvironment. We measured the release of IFNgamma and TNFalpha from tumour cell lines and PBMCs via ELISA in single culture, indirect co-culture separated by a semi-permeable membrane (1 µm pores), or in direct co-culture with cell-cell contacts. The experiments were performed with the HL cell line L428, as these cells have a marked downregulation of the MHC complex and are thus unlikely to provoke a mixed lymphocyte reaction (MLR) [10]. We also performed experiments, in which we cultured the human cell lines Daudi, MEC1, 293T and HT-29 separately, in indirect and in direct co-culture with PBMCs. Interestingly, only the HL cell lines L428 and L540 showed high IFNgamma secretion, which was up-regulated in the setting of direct PBMC co-culture, indicating that effects were HL-specific and not only a consequence of alloreactivity (Suppl. Fig. S2A).

**Figure 2:**
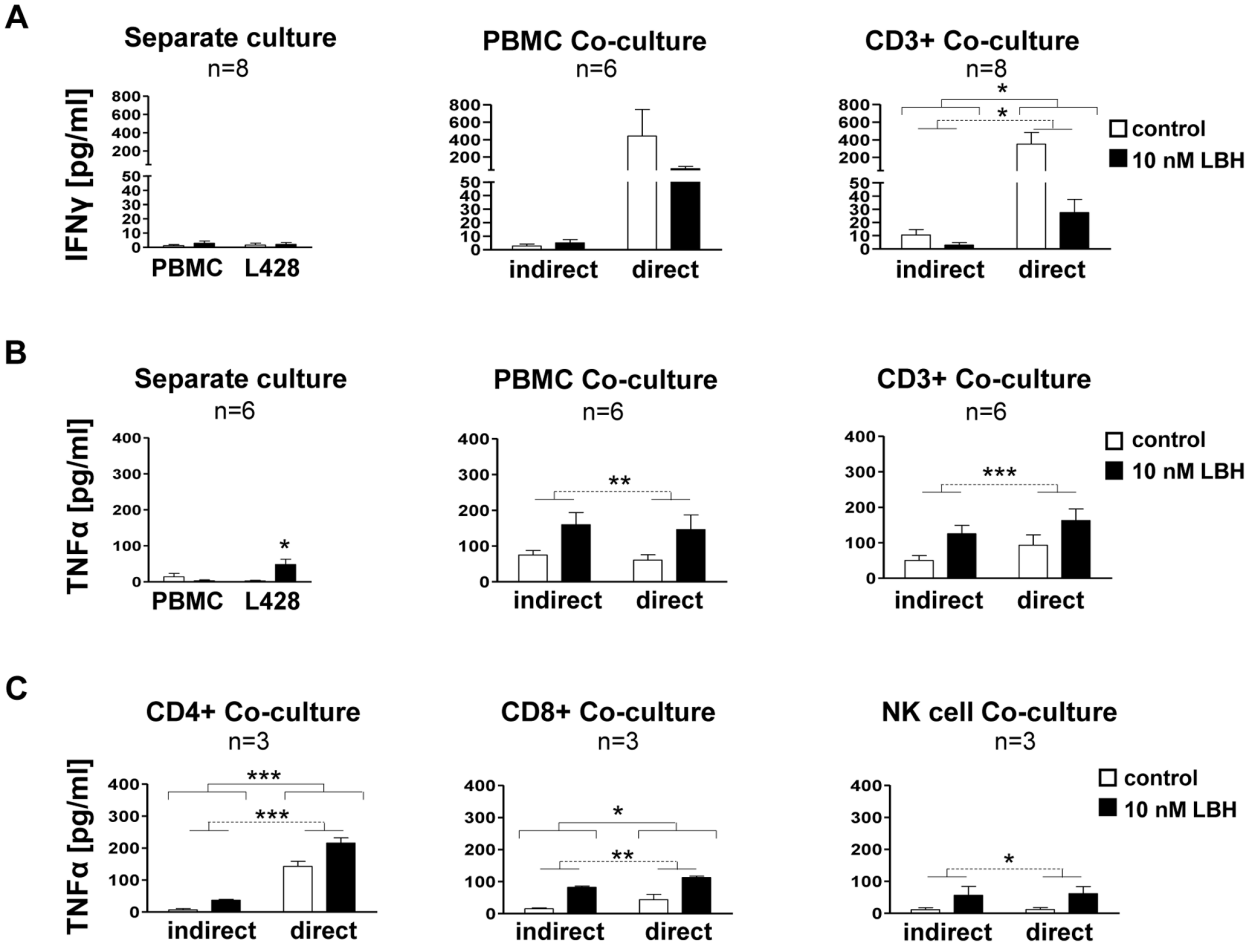
LBH589 modulated lymphocyte secretion of cytokines. (**A**) ELISA for IFNgamma from supernatants of cultured cells. Left panel: PBMCs and L428 cells were cultured separately. L428 cells were co-cultured with PBMCs (middle panel) and CD3+ cells (right panel; t = 36 hours). In indirect co-cultures, cells were separated by the membrane. The ratios of effector to tumour cells were 1∶1 with PBMCs and 1∶2 with CD3+ cells. PBMC/L428 co-culture did not show significant results due to high intra-group variance. For CD3+ cells, a significant IFNgamma increase in direct co-culture compared with indirect co-culture (p = 0.0198) as well as a significant downregulation upon LBH589-treatment (p = 0.0192) was observed (two-way ANOVA analysis; solid lines indicate the comparison of indirect versus direct co-culture; dashed lines the effect of LBH589 [also in B) and C]). (**B**) ELISA for TNFalpha and settings like in A). For both, PBMC/L428 and CD3+/L428 co-cultures, the effect of indirect vs. direct co-culture was not significant (p = 0.6637 and p = 0.3187, respectively), however the LBH589 treatment increased the TNFalpha secretion significantly (middle and right subpanels; p = 0.0059 and 0.0006, respectively; two-way ANOVA analysis). (**C**) ELISA for TNFalpha release in lymphocyte/L428 co-cultures. The ratio of effector to tumour cells was 1∶4 with CD4+ and CD8+ cells each and 1∶10 in NK cell co-cultures. The ratio of effector to tumour cells was 1∶4 with CD4+ and CD8+ cells each; and 1∶10 in NK cell co-cultures. LBH589 increased significantly the TNFalpha secretion in co-cultures of L428 with CD4+, CD8+ and NK cells (p = 0.0001, p = 0.0017 and p = 0.0253, respectively). The direct versus indirect co-culture method showed a significant impact on CD4+ (p = 0.0006) and CD8+ lymphocytes (p = 0.0328; two-way ANOVA analysis). The ratio of effector to tumour cells was 1∶4 with CD4+ and CD8+ cells each and 1∶10 in NK cell co-cultures.

IFNgamma was mainly secreted in direct co-cultures of L428 cells with PBMCs or CD3+ lymphocytes (Fig. 2A), indicating dependency on direct cell-cell contacts. A co-culture of L428 with different ratios of CD3+ cells indicated, that the T cells are the source of IFNgamma secretion but not the L428 cells (Suppl. Fig. S3A). Then we tried to identify which lymphocyte subset secreted IFNgamma. However, the co-cultures of L428 cells with CD4+, CD8+ or NK cells did not result in the release of detectable IFNgamma and remained below the detection limit of the ELISA (n = 3 each; data not shown). Thus, the small amounts of IFNgamma detected in PBMC cultures (Fig. 2A, left and middle sub-panels) are likely attributable to the natural variation in PBMC donors. In addition, this also indicates that the interplay of helper and cytotoxic T cells is mandatory for effective IFNgamma release. Importantly, IFNgamma release by CD3+ cells was significantly suppressed by LBH589 treatment (Fig. 2A).

When PBMCs and L428 cells were cultured separately, TNFalpha was released by both at a very low constitutive level (Fig. 2B, left sub-panel). However, when L428 cells were co-cultured with PBMCs in co-cultures, TNFalpha was secreted at high levels and even further boosted by LBH589 (Fig. 2B, middle sub-panel). When lymphocyte subsets were investigated, CD4+ and CD8+ cells showed a significant dependence on cell-cell contacts in direct co-cultures for high TNFalpha secretion, while the other tested subsets (CD3+, NK cells) demonstrated release in both, direct and indirect co-cultures, which indicated independence from cell-cell contacts.

Notably, LBH589 treatment significantly increased TNFalpha secretion in all tested lymphocyte co-cultures (Fig. 2B/C). When the effector to target cell ratio was changed, there was no significant change in the release of TNFalpha in the direct co-cultures (Fig. S3B in File S1. These data on LBH589's modulation of the cytokine network are complementary to a previous report on altered cytokine secretion (IL-5, IL-10 and IL-13) upon SAHA treatment [11]. We did not observe *in vitro* proliferation of PBMCs with or without LBH589 treatment (Fig. S2B in File S1).

**Figure 3:**
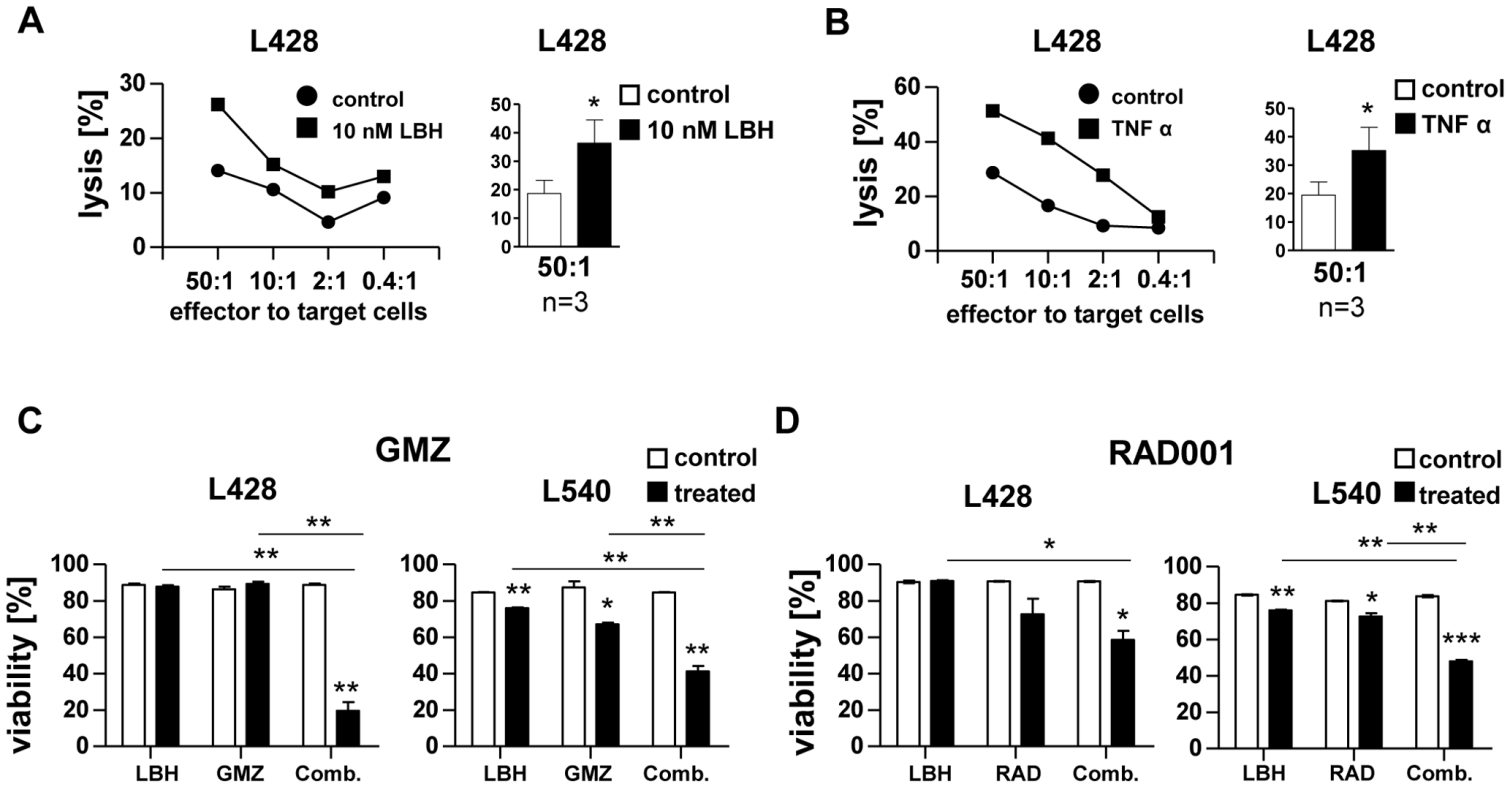
LBH589 enhanced cytotoxicity and worked synergistically with gemcitabine. (**A/B**) Killing assays in which target cells and PBMCs were pre-treated for 4 h with (**A**) 10 nM LBH or (**B**) 200 ng/ml TNFalpha. The panels show one representative experiment of three. The ratios of effector to target cells are indicated below the axis. In addition, the summary bar graph for the effector to target cell ratio 50∶1 of three independent experiments is given for both experimental settings (paired, two-sided t-test). (**C/D**) Combination regimens were tested with sublethal doses adjusted to each compound and cell line. L428 cells and L540 cells were treated with 10 nM LBH589 (LBH) and (**C**) Gemcitabine (GMZ; 500 ng/ml for L428 and 0.5 ng/ml for L540; n = 3) or (**D**) Everolismus (RAD; 5 µM for L428; 0.5 µM for L540; n = 3 each; paired, two-sided t-test).

Next we investigated the cytolytic activity of effector cells. Cytotoxicity was enhanced when both, target cells and PBMCs were pre-incubated with LBH589 (Fig. 3A). When PBMCs but not target cells were pre-incubated with LBH589 (and *vice versa*), killing assays also resulted in improved target cell killing (Figures S4A and B in File S1, respectively). Since TNFalpha release from all tested HL cell-lymphocytes co-cultures was increased upon LBH589 treatment (Fig. 2B/C), we added recombinant TNFalpha protein to a killing assay and observed an increased cytotoxic activity (Fig. 3B). Thus both, the increased MICA/B expression on HL target cells and the enhanced TNFalpha secretion, might contribute to the improved effector cell killing activity in response to LBH589 treatment.

We (Fig. S5A–C in File S1) and others established that LBH589 induced a dose-dependent (and also caspase-independent) decrease of cell viability in the HL cell lines L428, L540 and KM-H2 but also in peripheral blood mononuclear cells (PBMCs), albeit to a lesser extent in the latter. Furthermore, autophagy was induced by LBH589, since HL cell lysates showed a significant increase for the autophagy marker LC3II in Western Blot analysis (Figure S5D/E in File S1). Cells also formed intracellular, LC3-positve punctae in immunocytochemistry (Fig. S5F in File S1). These data are in line with previous studies of LBH589 inducing apoptosis and autophagy in HL cell lines [4] and may further contribute to an enhanced susceptibility against effector cell killing.

A study on the cytostatic drug gemcitabine (GMZ) revealed clinical efficacy in lymphomas as a mono-therapeutic [12] and was included in a clinical combination therapy study for HL patients [13]. In our experiments, a combination of low-dose LBH589 and GMZ showed a highly synergistic cytotoxic effect on HL cell lines (Fig. 3C). The combination was even more efficient in HL cell killing than the combination of LBH589 and the mTOR inhibitor RAD001 (everolismus) (Fig. 3D), which was recently reported [4]. In contrast, combination with Brentuximab-Vedotin was not significantly efficient (data not shown) due to downregulation of CD30 as a target (Fig. 1B/C).

Taken together, our data reveal an impact of the HDACi LBH589 on non-malignant cells present in the HL microenvironment such as an increased effector cell killing activity that might be induced by the upregulation of MICA/B in target cells and an enhanced TNFalpha release. The potential relevance of TNFalpha in oncological treatment was supported by a study in which GMZ and intra-tumoural injections of TNFalpha were successful [14], while in contrast anti-TNFalpha therapies can lead to the emergence of lymphomas [15].

## Materials and Methods

### Ethics statement

Collection of and experiments with blood samples of healthy donors were approved by the local ethics committee of the University of Cologne under reference number11-140 and donors provided written consent.

### Cells and reagents

Primary blood mononuclear cells (PBMCs) were isolated from buffy coats by Ficoll-Plaque density gradient centrifugation. Lymphocyte isolation was performed with isolation Kits and the VarioMACS System (all from Miltenyi, Bergisch-Gladbach, Germany). The purity of isolated lymphocyte subpopulations was tested via flow cytometry on control samples. NK cells were isolated via negative selection (NK cell isolation kit; Cat# 130-092-657; Miltenyi; >99% purity). CD3+ (>97% purity), CD4+ and CD8+ cells were isolated via positive selection with MicroBeads (CD3/CD4/CD8 MicroBeads, human; Miltenyi; Cat# 130-050-101, 130-045-101 and 130-045-201, respectively). CD3− cells were obtained via depletion of CD3+ cells with a purity of >96%.

The cell lines L428, L540, Daudi, MEC1 and 293T were purchased from the DSMZ (Deutsche Sammlung von Mikroorganismen und Zellkulturen GmbH (DSMZ, Braunschweig, Germany) and HT-29 cells from the American Type Culture Collection (ATCC, Manassas, VA, USA). Cells were cultivated in RPMI-1640 with 10% FBS and antibiotics. Panobinostat (LBH589) and everolismus (RAD001) were generously provided by Novartis (Basel, Switzerland) and gemcitabine (GMZ) (Sigma-Aldrich Chemie GmbH, Taufkirchen/Munich, Germany). Tables S1 and 2 in File S1 give further details on antibodies, and kits & reagents, respectively.

### Flow cytometric analysis of apoptosis

Cells were washed, resuspended in annexin-binding buffer and incubated with Annexin V-FITC and 7-amino-actinomycin D (7-AAD). Flow cytometry for cell surface markers: Cells were pre-treated and incubated with the corresponding antibodies. In case of unconjugated antibodies, cells were washed and incubated with a secondary conjugated antibody. Analysis was performed with a BD FACSCalibur cytometer (Becton Dickinson GmbH, Heidelberg, Germany) and the software Cellquest (Beckton Dickinson) and FlowJo (Tree Star Inc., Ashland, OR, USA).

### Co-culture experiments

PBMCs and tumour cells L428 were seeded into 24 well plates and stimulated with LBH589 or vehicle either in separate wells, or indirect co-cultures with a semi-permeable membrane insert, or in direct co-cultures with cell-cell contacts. After 36 hours, supernatants were taken, purified and kept frozen until analysis.

### Immunofluorescence microscopy

Cells were spun on microscopy slides with a cytospin device and fixed with PFA-containing Fixation buffer for 10 minutes at room temperature. Cells were permeabilised for 10 minutes with ice-cold Methanol (70%), and blocked with 10% FBS in PBS followed by antibody staining. Immunofluorescence microscopy was carried out with the AxioScope A1 microscope and an MRm camera (Zeiss, Jena, Germany).

### Western Blotting

Cells were treated for the duration 0 or 24 hours with 100 nM LBH or vehicle control as well as 100 nM Bafilomycin A1 to prevent degradation of autophagosomes. Cells were lysed with RIPA buffer including protease inhibitor cocktail, debris removed by centrifugation (4°C, 10000×g, 10 min) and clear lysate stored frozen until use. Protein concentration was determined with a BCA-assay. 5 µg protein were mixed with Laemmli buffer, denatured for 5 min at 95°C, loaded onto SDS-PAGE gels (15%) and blotted via wet transfer onto methanol-activated PVDF-membranes. Antibodies are listed in Table S1 in File S1. An ECL kit was used for detection on x-ray films. Films were scanned and densiometric analysis performed with ImageJ according to the guidelines by Dr Luke Miller (www.lukemiller.org).

### Co-culture experiments

Co-culture experiments were carried out with 5×10^4^ L428 target cells and appropriate numbers of lymphocytes to match the ratios of lymphocytes to target cells. Different ratios as shown in Fig. S1A in File S1 are indicated below the axis. In indirect co-cultures, the target cells were seeded into inserts with pores of 1 µM diameter.

### ELISA

TNFalpha and IFNgamma were measured with commercial kits and CD30 was quantified according to a previously published protocol [16].

### Cytotoxicity assay

Procedures were based on a previously described protocol [17]. In short, L428 target and/or PBMCs were separately pre-incubated with 10 nM LBH589 for 4 hours, then taken up in hypotonic Europium labelling buffer (Eppendorf, Hamburg, Germany) with added 10 mM DTPA and 2 mM EuCl final concentration. Labelling was done in 4 mm cuvettes in the Multiporator electroporator (Eppendorf) at 180 V for 80 µs. After three times washing, cells were seeded into 96 well plates (round bottom) together with PBMCs. Each experimental reading point was done in triplicate. Baseline release remained untreated and maximum release stimulated via RPMI+1% Tritin-X100. When rec. TNFalpha was used, it was also added at the beginning of the final incubation time (200 ng/ml). After 3 h incubation at 37°C, the plate was centrifuged (∼130×g for 5 min) and 20 µl supernatant transferred into a fresh 96 well plate (flat bottom). After adding 200 µl/well enhancement solution, the plate was read on a Wallac Victor^2^ 1420 multilabel counter plate reader (Perkin Elmer, Waltham, MA, USA). Data were analysed with MS-Excel and GraphPad Prism software.

### RT-PCR

Total RNA was isolated from 10^7^cells with the RNeasy Mini QIAshredder Kit (QIAGEN, Hildesheim, Germany) according to the manufacturer's instructions, the RNA quantity and concentration determined with the ND-1000 spectrophotometer (Nanodrop Technologies Inc., Wilmington, DE, USA), and 2 µg RNA was reverse transcribed with the Superscript III Reverse Transcriptase Kit (Invitrogen, Life Technologies GmbH, Darmstadt, Germany) with Oligo dT primers according to the manufacturer's instructions. Primer sequences were as follows: CD30-primer 5′-GCGAGAAGCTTATGC GCGTCCTCCTCGCCGCG-3′, and 5′-AGTGACGGATCCACTTACCTGTTGATGAG TATTTATGATAACAAAGT-3′, as well as standard primer for GAPDH. Standard PCR with *Taq* DNA Polymerase (Fermentas GmbH, Leon-Rot, Germany) was performed on a thermocycler T3000 (Biometra GmbH, Göttingen, Germany) with the programme 94°C for 1 min., 30 cycles of 94°C for 45 sec., 60°C for 30 sec. and 72°C for 2 min, and finally 72°C for 7 min.

### Statistics

GraphPad Prism Software (San Diego, CA, USA) was used to calculate statistics. In figures, p-values are indicated as * for p≤0.05, ** for p≤0.01, and *** for p≤0.001. If not stated otherwise, bar charts show mean +/− SEM and a two-sided t-test used.

## Supporting Information

File S1 **Contains Figures S1–S5 and Tables S1–S2.**
(DOCX)
