# Supplementary material for: The Histone Deacetylase Inhibitor LBH589 (Panobinostat) Modulates the Crosstalk of Lymphocytes with Hodgkin Lymphoma Cell Lines
Source: PLoS One. 2013 Nov 21;8(11):e79502. doi: 10.1371/journal.pone.0079502 (PMC3836980; doi:10.1371/journal.pone.0079502)
Supplement: File S1 — Contains Figures S1–S5 and Tables S1–S2. (DOCX) [file pone.0079502.s001.docx]

**Supplementary Materials**

**Supplementary Figure S1**

**
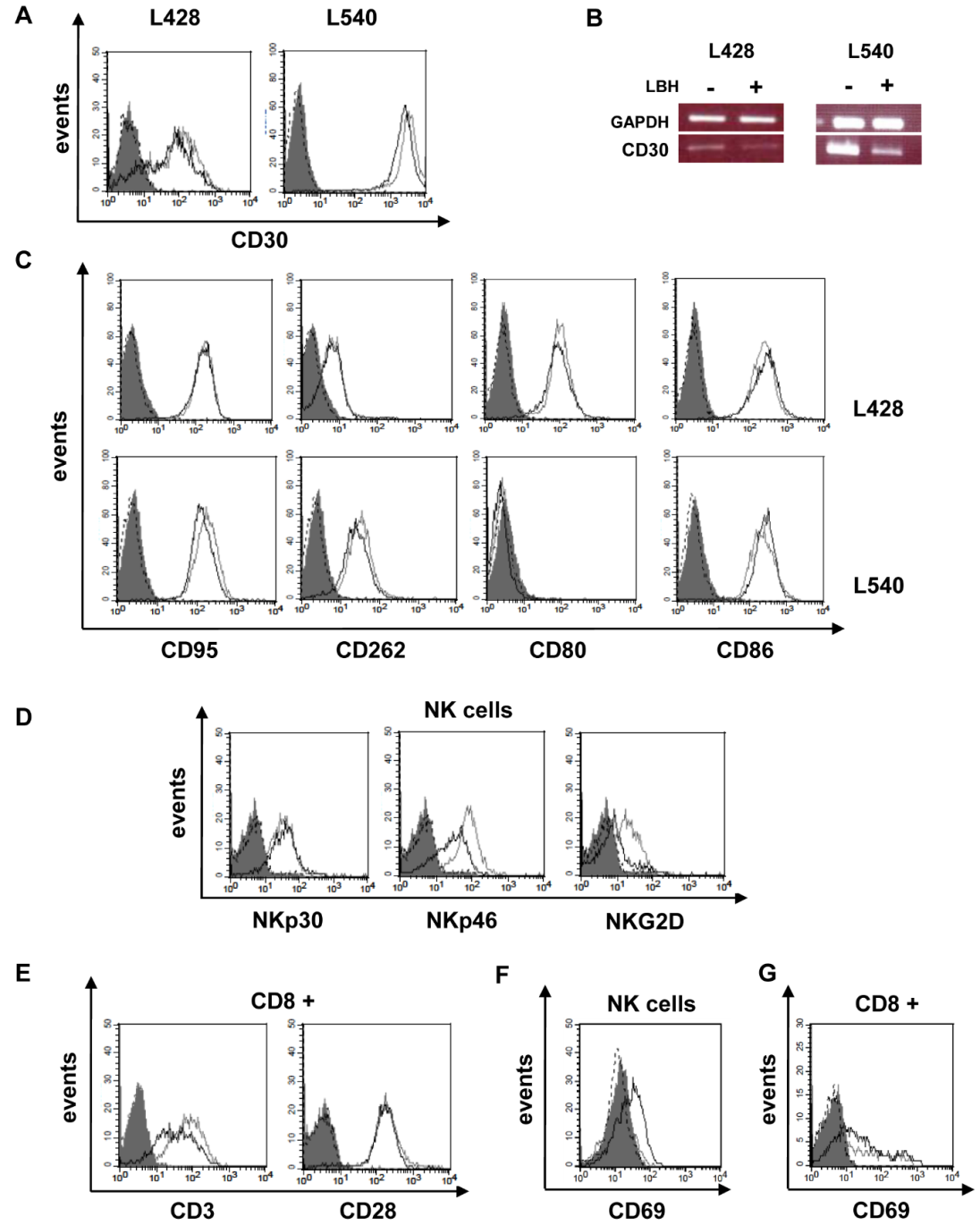
**

**Supplementary Figure S2**

**
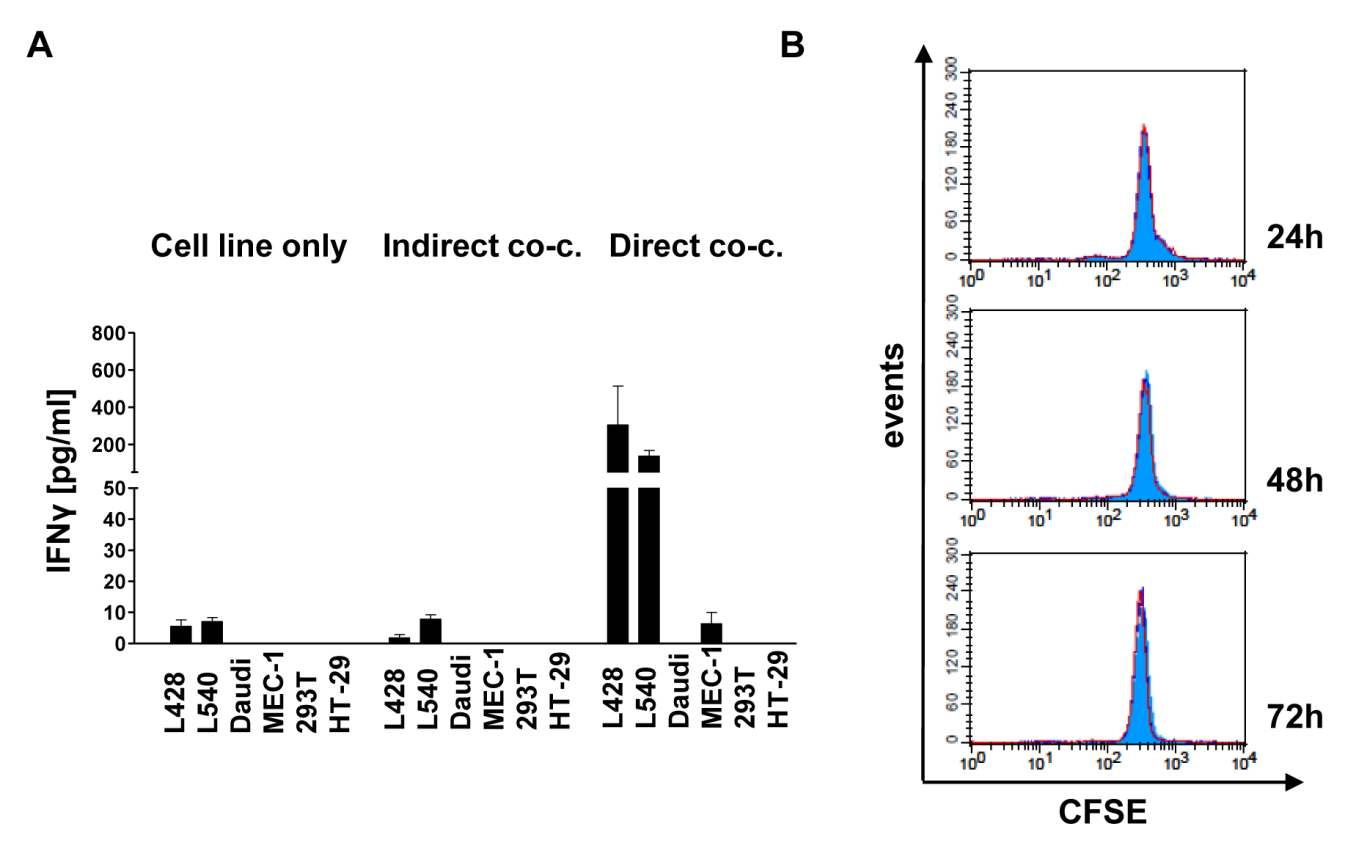
**

**Supplementary Figure S3**

**
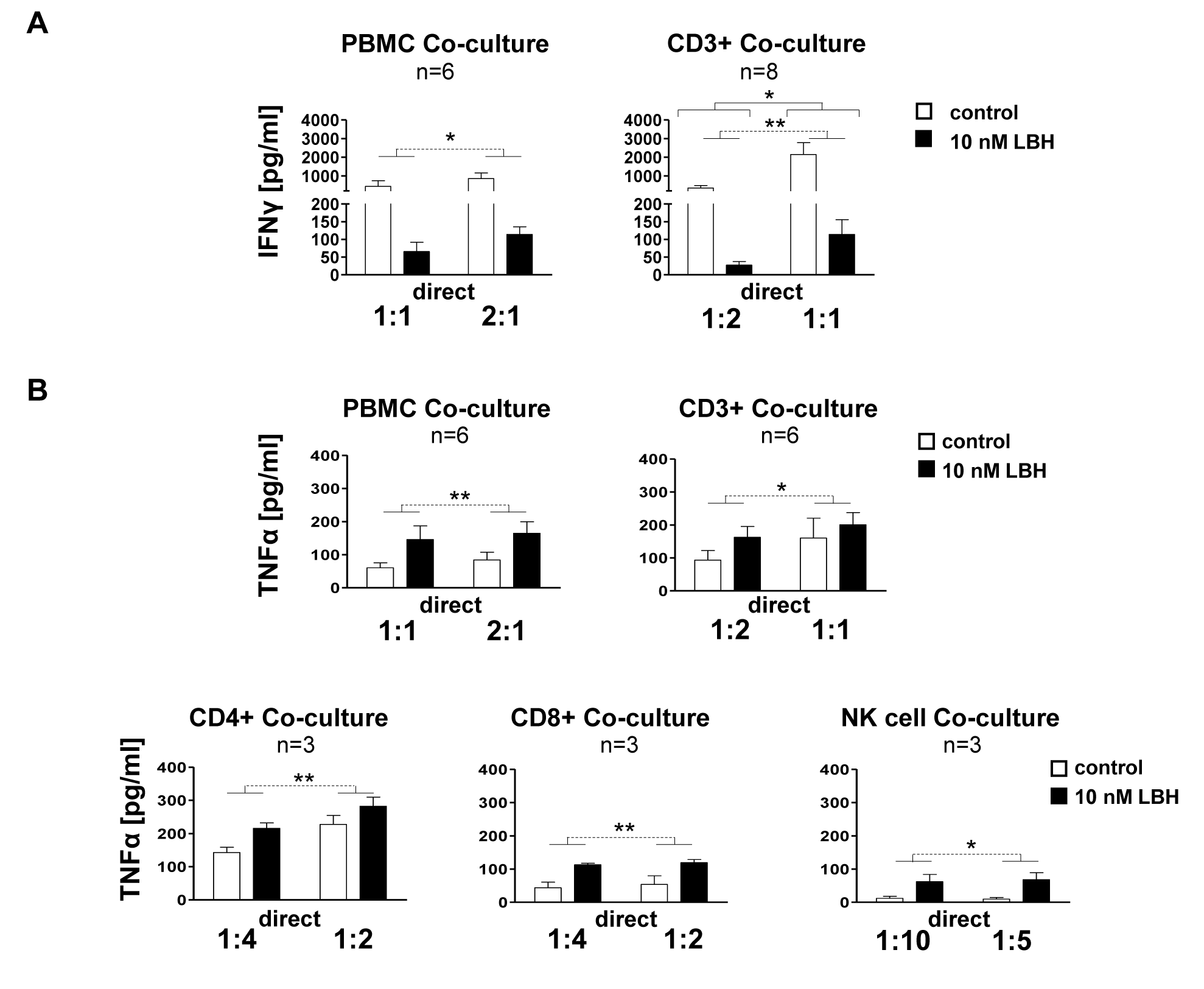
**

**Supplementary Figure S4**

**
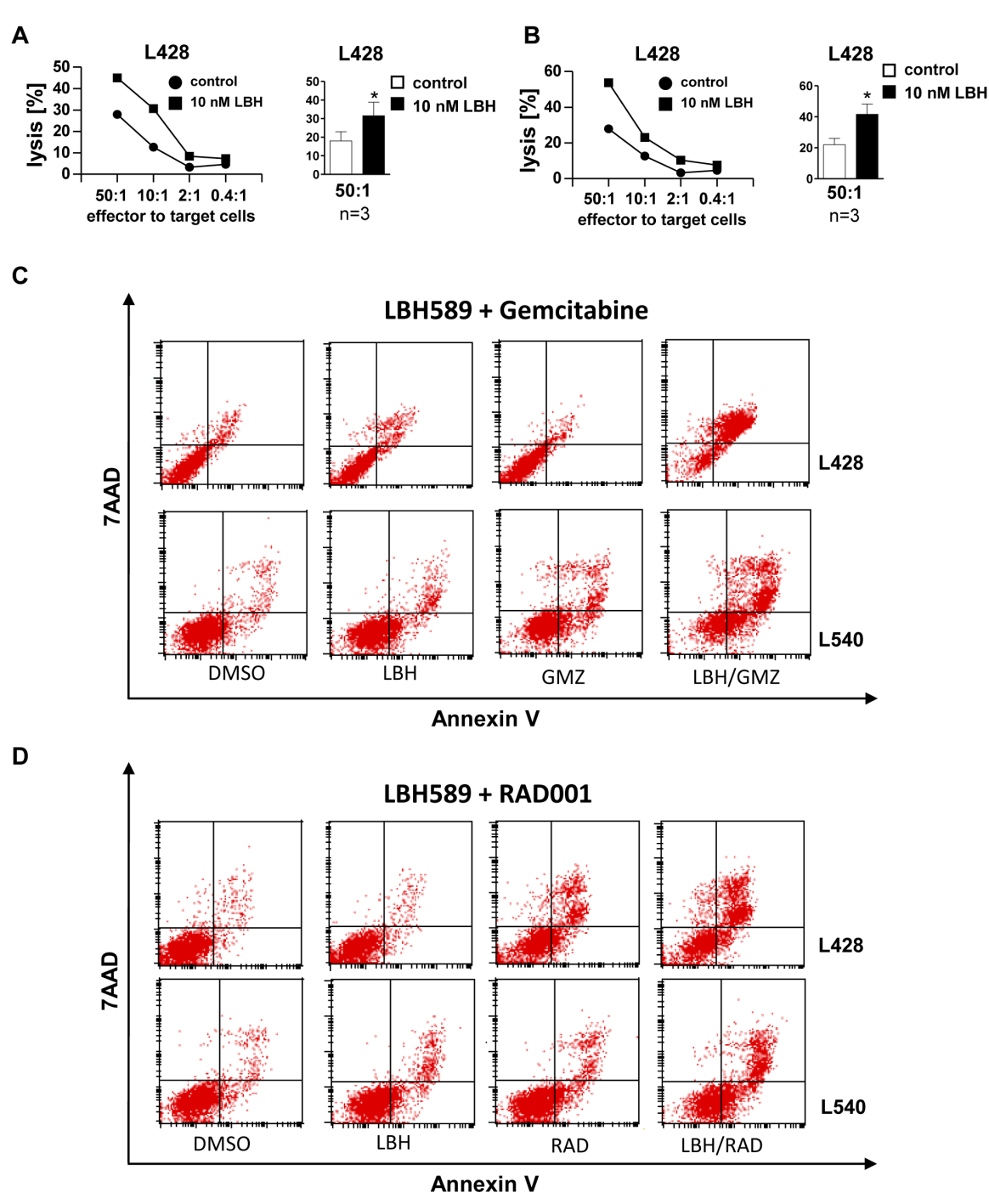
**

**Supplementary Figure S5**

**
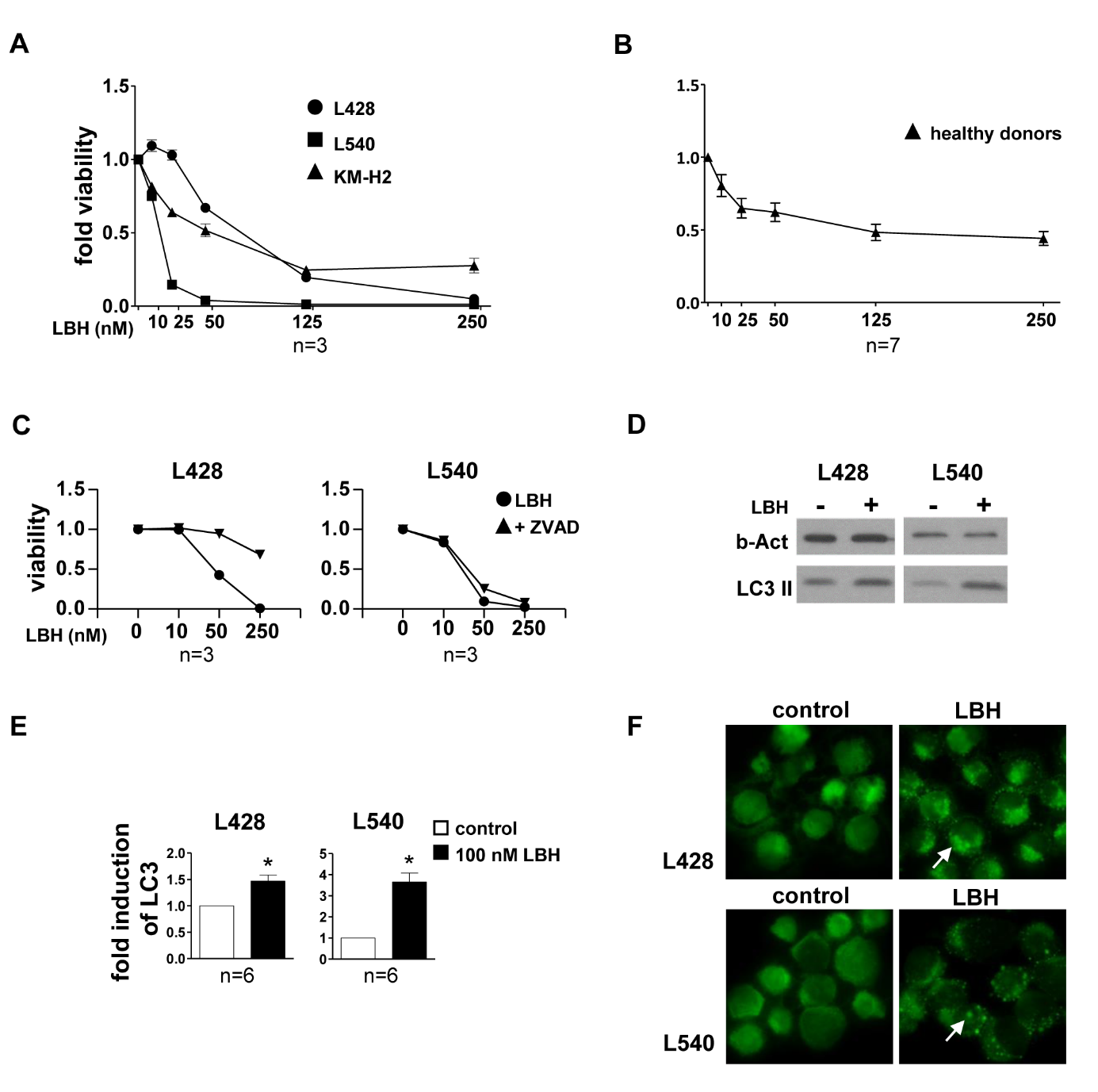
**

**Supplementary Table S1**

| **Antibody** | **Application** | **Dilution** | **Vendor** | **Catalogue no.** |
| --- | --- | --- | --- | --- |
| LC3 | WB | 1:500 | NB | NB100-2220 |
| LC3-FITC | IF | 1:200 | NB | NB100-2220F |
| Actin | WB | 1:4000 | S-A | A1978 |
| anti-Mouse-HRP | WB | 1:10000 | DN/JIT | 115-035-003 |
| anti-rabbit-POD | WB/ELISA | 1:10000 | DN/JIT | 111-035-003 |
| MICA/B-A647 | FACS | 1:50 | BL | 320914 |
| Annexin V-PE | FACS | 1:50 | BD | 556422 |
| 7-AAD | FACS | 1:100 | BL | 420404 |
| CD69-FITC | FACS | 1:50 | BL | 310904 |
| CD95 | FACS | 1:50 | BD | 555674 |
| CD262 | FACS | 1:50 | BL | 307405 |
| CD80 | FACS | 1:50 | BL | 305208 |
| CD86 | FACS | 1:50 | BD | 555665 |
| BAG6 | FACS | 1:50 | produced in our lab | 3E4 |
| CD30 | FACS | 1:50 | BD | 550041 |
| NKG2D-FITC | FACS | 1:50 | abcam | Ab35035 |
| NKp30-PE | FACS | 1:50 | BL | 325208 |
| NKp46-PE | FACS | 1:50 | BL | 331908 |
| goat-anti-mouse-PE | FACS | 1:50 | BL | 405307 |
| Isotype control IgG1-PE | FACS | 1:50 | BL | 400114 |
| Isotype control IgG1-FITC | FACS | 1:50 | BL | 556649 |

**Supplementary Table S2**

| **Reagent /Chemical /Kit** | **Application** | **Vendor** |
| --- | --- | --- |
| Iscove’s Modified Dulbecco’s Medium (IMDM) | Cell culture | LT |
| Dulbecco’s Modified Eagle Medium (DMEM) | Cell culture | LT |
| RPMI1640 | Cell culture | LT |
| Fetal bovine serum (FBS) Gold | Cell culture | PAA |
| Penicillin/Streptomycin, 100x | Cell culture | PAA |
| LSM1077 Lymphocyte separation medium | PBMC purification | PAA |
| Bafilomycin A1 | Cell culture experiment | S-A |
| Complete protease inhibitors | Cell lysis buffer, WB | Ro |
| BCA Protein Assay Kit | Protein quantification | PBT |
| 5 x Laemmli buffer | WB | self-made |
| BioTrace PVDF membrane | WB | Pall |
| enhanced luminescence detection kit | WB | PBT |
| Pacific Blue x-ray film | WB | SC |
| Human TNFalpha ELISA MAX Standard Set | ELISA | BL |
| Human IFNgamma ELISA MAX Standard Set | ELISA | BL |
| 1-Step Ultra TMB-ELISA | ELISA | PBT |

**Supplementary References**

**1. Bosshart H, Jarrett RF. (1998) Deficient major histocompatibility complex class II antigen presentation in a subset of hodgkin's disease tumor cells. Blood 92: 2252-2259.**

**2. Lee SP, Constandinou CM, Thomas WA, Croom-Carter D, Blake NW, et al. (1998) Antigen presenting phenotype of hodgkin reed-sternberg cells: Analysis of the HLA class I processing pathway and the effects of interleukin-10 on epstein-barr virus-specific cytotoxic T-cell recognition. Blood 92: 1020-1030.**

**3. Pollack MS, Heagney SD, Livingston PO, Fogh J. (1981) HLA-A, B, C and DR alloantigen expression on forty-six cultured human tumor cell lines. J Natl Cancer Inst 66: 1003-1012.**

**4. Stacchini A, Aragno M, Vallario A, Alfarano A, Circosta P, et al. (1999) MEC1 and MEC2: Two new cell lines derived from B-chronic lymphocytic leukaemia in prolymphocytoid transformation. Leuk Res 23: 127-136.**

**5. Vogel R, Al-Daccak R, Drews O, Alonzeau A, Mester G, Charron D, Stevanovic S, Mallet J. (2013) Mass Spectrometry Reveals Changes in MHC I Antigen Presentation After Lentivector Expression of a Gene Regulation System. Mol. Ther. Nucleic Acids 12;2:e75.**

**Legends**

**Supplementary Figure S1: Cell ratio-dependent TNFalpha release in co-cultures and LBH589 enhanced cytotoxicity in killing assays**

**(A)** One representative of three independent flow cytometry experiments is shown, in which CD30 was detected on the surface of L428 and L540 cells with and without previous LBH589 treatment (48 h, 20 nM). Dark grey = isotype control, 0 nM ; dashed line = isotype control, 20 nM; solid grey line = specific AB, treatment with 0nM LBH589; black solid line = specific AB, treatment with 20 nM LBH589. The summary bar graph is given in Fig. 1B. **(B)** RT-PCR for CD30 expression, as well as GAPDH control, in L428 and L540 cell lines treated with 20 nM LBH589 for 48h. One representative result out of three independent experiments is shown. **(C)** One representative of three independent flow cytometry experiments is shown, in which CD95, CD262, CD80 and CD86 were detected on the surface of L428 and L540 cells via flow cytometry with previous LBH589 treatment vs. control (36 h, 20 nM). Legend of treatments here and for other panels in this figure: as in Suppl. Fig 1A. **(D)** One representative of at least three independent flow cytometry experiments is shown, in which the activating receptors NKp30, NKp46 and NKG2D were detected on the surface of primary NK cells of healthy donors with previous treatment with LBH589 vs. control (24 h, 20 nM). The summary bar graph is given in Fig. 1D. Legend of treatments: as in Suppl. Fig 1A.

**(E)** One representative of at least three independent flow cytometry experiments is shown, in which the activating receptors CD3 and CD28 were detected on the surface of primary CD8+ T cells cells of healthy donors with previous treatment with LBH589 vs. control (24 h, 20 nM). The summary bar graph is given in Fig. 1E.

**(F)** One representative of at least three independent flow cytometry experiments is shown, in which the surface marker CD69 for the activation status of the cells was detected on the surface of primary NK cells and T cells of healthy donors with previous treatment with LBH589 vs. control (24 h, 20 nM).

**Supplementary Figure S2: IFNgamma secretion by human cell lines and proliferative activity of PBMCs**

**(A)** ELISA for IFNgamma was performed with supernatants from culture experiments. The human Hodgkin lymphoma cell lines L428 and L540 Daudi (B cell Burkitt Lymphoma), MEC1 (B cell-CLL), 293T (human embryonic kidney) and HT-29 (human colon carcinoma) were cultured either alone or in co-culture. Co-culture was indirect by separation with an insert with a membrane, or in direct co-culture with cell-cell contact. L428 cells were reported to have MHC II molecules on the surface [1] and very low levels of MHC I molecules [2]. Daudi are described as MHC I-negative but as MHC II-positive [1,3], while MEC1 cells were investigated only for the presence of HLA-DR [4]. 293T cells [5] and HT-29 cells [3] are positive for HLA-A expression. IFNgamma, a potential indicator of mixed lymphocyte reaction (MLR), was only elevated in the direct co-culture with L428 and L540 but not with the other cells. This suggests that the secreted IFNgamma was released by a mechanism other than MLR, as an IFNgamma release via MLR should have also occurred in the direct co-culture with the other cell types. n=9 for L428 cell cultures and n=3 for the other cell lines.

**(B)** Primary PBMCs of one donor in two independent experimental runs were labelled with CFSE, stimulated with 0, 10 and 20 nM LBH589 and subjected to flow cytometry after 24, 48 and 72 hours. The histograms indicate equal distribution of CFSE in a single cell population, thus indicating the absence of proliferation. Filled blue area = 0 nM, light blue line = 10 nM and red line = 20 nM.

**Supplementary Figure S3: IFNgamma and TNFalpha secretion in lymphocyte-HL cell co-cultures**

**(A)** ELISA for IFNgamma was performed with supernatants from L428 co-culture experiments with PBMCs and CD3+ cells (n=6 and n=8 respectively). Different ratios of effector to target cells are indicated below the axis. The dashed lines indicate significant effects upon LBH589 treatment, while solid lines indicated significant effects due to different effector to target cell ratios (two-way ANOVA analysis). Results of direct co-cultures are shown. A ratio-dependent increase in cytokine secretion was only significant in the CD3+ co-culture (p=0.0152).

**(B)** ELISA for TNFalpha from L428 co-cultures with PBMCs, CD3+, CD4+, CD8+ or NK cells (t=36 hours). Two different ratios of effector to tumour cells were used for each setting. Ratios are indicated below the axis. The impact of LBH589 treatment was significant in all co-culture experiments, while the different ratios of effector to target cells had no significant impact (two-way ANOVA analysis). The dashed lines indicate significant effects upon LBH589 treatment. (two-way ANOVA analysis). Bar charts in this figure indicate mean + S.E.M.

**Supplementary Figure S4: LBH589 enhanced cytotoxicity and worked synergistically with gemcitabine**

**(A/B)** Killing assays with PBMCs effector and L428 target cells. Either the PBMCs **(A)** or the target cells only **(B)** were pre-incubated with LBH589 prior to the killing assay (4h pre-incubation time; n=3 each). Shown are one representative experiment and the bar chart summary of three independent experiments. In all cases the pre-incubation enhanced the killing efficacy (two-sided, paired t-test). Since these experiments were done simultaneously as those presented in Fig. untreated control group

**(C)** Combination regimen experiments. Cells were treated with compounds and after LBH589 and gemcitabine (GMZ) – each at sublethal doses, adjusted to the cell line - were combined to treat L428 and L540 cells. While the summary is presented in Figure 3C, here representative dot blot diagrams are shown. The diagrams show a presumably synergistic toxic effect on the HL cells, as the number of dots is increased in the two upper quadrants upon treatment. Doses: GM) was used at 500 ng/ml for L428 and at 0.5 ng/ml for L540, while LBH589 at 10 nM. One of three experiments is shown.

**(D)** Combination regimen experiments. LBH589 and Everolismus (RAD001) were combined to treat L428 and L540 cells. While the summary is presented in Figure 3D, here representative dot blot diagrams are shown. The diagrams show a synergistic toxic effect on the HL cells, as the number of dots is increased in the two upper quadrants upon treatment. Doses: RAD was used at 5 µM for L428 and at 0.5 µM for L540, while LBH589 was used at 10 nM. One of three experiments is shown.

**Supplementary Figure S5: LBH589 enhanced cell death and autophagy in HL cells**

**(A-C)** Cell death was measured via 7-AAD flow cytometry after 48 hours exposure to different doses of LBH589 in **(A)** HL cell lines L428, L540 and KM-H2 (n=3 independent experiments), **(B)** primary PBMCs (n=7 donors) and **(C)** HL cell lines L428 and L540 (n=3) with the pan-caspase inhibitor zVAD-fmk (50 µg/ml) after 48h incubation.

**(D-F)** Autophagy was detected via Western blotting for LC3II; representative images are shown **(D)** and six experiments were summarised for normalised densiometric measurements **(E)**. Cells were treated with 100 nM LBH589 and 100nM Bafilomycin A1 (for accumulation of autophagosomes) for 24 hours. A two-sided Wilcoxon rank test showed p=0.03 for both cell lines. **(F)** Fluorescence immunocytochemistry for staining LC3I/II indicated autophagy induction upon LBH-treatment (100 nM for 24h). White arrows indicate punctae or autophagosomes. One representative experiment of three is shown.

**Supplementary Table S1: Antibodies used in Experiments**

*Abbreviations:* **APC** allophycocyanin, **FITC** fluorescein isothiocyanate, **HRP** horseradish peroxidase, **IF** immunofluorescence, **PE** phycoerythrin, **WB** Western Blotting

*Abbreviations of vendors:* **abcam** (Cambridge, UK), **BC** Beckman Coulter, **BL** BioLegend (San Diego, CA, USA), **DN** Dianova (Hamburg, Germany), **JIT** Jackson ImmunoResearch Laboratories (West Grove, PA, USA), **NB** Novus Biologicals **S-A** Sigma-Aldrich (Germany).

**Supplementary Table S2: Reagents, kits and chemicals**

*Abbreviations:* **BL** BioLegend (San Diego, CA, USA), **LT** life technologies (Darmstadt, Germany),**PAA** (Linz, Austria), **Pall** Corporation (Pensacola, FL, USA), **PBT** Pierce Biotechnology / Thermo Scientific (Rockford, IL, USA), **Ro** Roche Diagnostics GmbH (Mannheim Germany), **S-A** Sigma-Aldrich (Munich, Germany), **SC** Santa Cruz Biotechnology (Santa Cruz, CA, USA).
